# Investigating barriers teachers face in the implementation of inclusive education in high schools in Gege branch, Swaziland

**DOI:** 10.4102/ajod.v7i0.391

**Published:** 2018-12-06

**Authors:** Sifiso L. Zwane, Matome M. Malale

**Affiliations:** 1 Ministry of Education and Training, Mbabane, Swaziland; 2 Department of ABET and Youth Development, University of South Africa, South Africa

## Abstract

**Background:**

The kingdom of Swaziland is a signatory to policies on universal education that ensure high quality basic education for all. Education for All is a commitment to provide equal opportunities for all children and the youth as provided for in the country’s constitution of 2005. The tone for the introduction of inclusive education in Swaziland was inevitably set by the new constitution of 2005. Since then several policies have been produced by the government, all aimed at providing equal education opportunities to all children in the country. These policies include the Swaziland National Children’s Policy (2009), Poverty Reduction Strategy and Action Plan (2006) and Draft Inclusive Education Policy (2008). The Education for All Policy (2010) is the policy that upon implementation became a stimulus for the introduction of inclusive education into mainstream schools; as a result, all teachers in the country’s schools were expected to be competent enough to teach learners with a wide range of educational needs. However, in-service teachers received inadequate staff development and training ahead of the implementation of inclusive education and a majority of teachers were not professionally developed for inclusive education, as pre-service students at tertiary training level.

**Objectives:**

This study investigated barriers in the implementation of inclusive education at high schools in the Gege branch, Swaziland, with a view to finding lasting solutions to inform research and government policy.

**Method:**

This research is a qualitative interpretive case study based on selected schools in the Gege branch of schools. Data was obtained through semi-structured research interviews and document analysis. It was processed and analysed through data coding, unitising, categorising and emergence of themes, which became the findings of the study.

**Results:**

Lack of facilities in the governments’ schools and teachers’ incompetence in identifying learners facing learning challenges in their classrooms are some barriers to inclusivity.

**Conclusion:**

The study concludes that there is a need for the Ministry of Education and Training to craft an inclusive curriculum in line with the inclusive policy in order to cater for the diverse educational needs of all learners in mainstream schools. It is thought that instituting a vibrant in-service and pre-service teacher training programme by the Ministry of Education and Training will increase teachers’ capacity to a level where teaching in inclusive classrooms does not negatively affect their competence.

## Introduction

According to Donald, Lazarus and Lolwana (2002:4) ‘barriers to learning’ refer to any factors, either internal or external to the learner, that cause a hindrance to that person’s ability to benefit from schooling. Researchers have found that implementation of inclusive education from policy to practice is often met by many barriers in countries where studies have been conducted (Donald et al. 2002:4). Swaziland is a signatory to policies on universal education that seek to ensure the provision of high quality basic education for all. Education for All is a commitment to providing equal opportunities for all children and youth as stipulated in its highest piece of legislation, with a view to affording equal education opportunities to all children in the country.

Several other international and local legislations have been signed by Swaziland. These policies commit the country to providing inclusive education in our school system, including the Salamanca Statement, Framework for Action on Special Needs (United Nations Educational, Scientific and Cultural Organisation [UNESCO] 1994). The constitution of the country states that every Swazi child within 3 years of the commencement of this constitution has the right to free education in public schools at least up to the end of primary school, beginning with first grade. Moreover the Swaziland Constitution (2005:25) stipulates that compulsory inclusive basic education be provided free for all children in the country, irrespective of gender, age, life circumstances, health and disability, stage of development, capacity to learn or financial circumstances.

By signing the aforementioned international and local policies, Swaziland pledged its legal commitment to implementing inclusive education in mainstream schools. The extent to which the Swaziland government has domesticated the aforementioned international inclusive education policies attests to and affirms the commitment of the Ministry of Education and Training to the provision of quality education for all learners in the country.

Not many studies have been conducted in Swaziland in relation to how effectively inclusive education has been rolled out to schools. However, the few studies that have been found do point out that the Ministry of Education and Training in the country is facing some challenges in implementing inclusive education effectively in mainstream schools. These studies include ‘Challenges in the implementation of inclusive education at Elulakeni cluster primary schools in Shiselweni district of Swaziland’ (2015) and ‘Supporting teachers to implement inclusive education in the Kwaluseni District, Swaziland’ (2012). Against this background the main problem of this study can be stated as to determine barriers in the implementation of inclusive education in schools in the Gege branch, Swaziland, in order to discover how best these barriers can be overcome. The problem has been investigated by, among other means, reviewing literature from related studies. The literature expounds on the pedagogical and curriculum factors, inappropriate assessment procedures, teacher training barriers to effective teaching and learning, unqualified and underqualified teachers, lack of support for teachers, and inappropriate teaching and learning methods and support material.

### Curriculum delivery barriers to learning

The curriculum and teaching methods used by educators play a pivotal role in as far as attaining effective teaching in inclusive classrooms is concerned. However, a rigid and inflexible curriculum that does not allow for individual differences can lead to learning breakdown (Motitswe 2012:39). Negative effects on education include aspects such as lack of relevance of subject content; lack of appropriate learning materials, resources and assistive devices; inflexible styles of teaching and classroom management; and inappropriate ways of assessing learning. Motitswe (2012:39) further notes that one of the most serious barriers to learning can be found within the curriculum itself and relates primarily to its inflexible nature. This prevents it from meeting diverse needs among learners; hence the curriculum should be adapted to suit all learners and the principle of learner-centeredness must also be taken into consideration.

The inflexible nature of the curriculum prevents it from meeting learners’ diverse needs. In research by Zimba (2011:53) at a pilot inclusive primary school, he discovered that the curriculum used at the school was not modified to accommodate learners with a wide array of educational needs. Teaching pupils with learning disabilities (LD) using mainstream techniques makes learning and teaching a challenge for the teacher and the learner in an inclusive class. For instance in a curriculum adapted for so-called normal learners, a teacher can teach pupils by writing on the board. However, this is not applicable in the case where some pupils cannot see. The use of concrete objects must therefore be emphasised in the inclusive curriculum for all learners to benefit (Zimba 2011:54).

### Inappropriate assessment procedures

*Assessment* refers to the ways teachers and other people involved in a pupil’s education systematically collect and then use information about that pupil’s level of achievement and/or development in different areas of their educational experience (academic, behaviour and social) (Watkins 2007:15). The central purposes of assessment are stated as providing information on learner achievement and progress and improving the process of learning and teaching (Department of Education 1998:4). Among some assessment procedures regarded as inappropriate are the following.

#### Aptitude achievement discrepancy

Batsche (2006) argues that although early studies suggested that a significant discrepancy between intelligence quotient (IQ) and achievement demarcated unexpected underachievement, this hypothesis has not held up. In the most extensive studies involving children with LD, comparisons of groups defined as IQ-discrepant and non-discrepant poor readers do not show meaningful differences in prognosis, cognitive skills related to the reading process (such as phonological awareness) or instructional response once definitional variability is accounted for in the models (Stuebing et al. 2002). Unlike Rutter and Yule (1975), these studies generally exclude children with intellectual developmental disorders and brain injuries, who accounted for the ‘poor reader’ group in that study. There are also major psychometric problems attributable to the small measurement error of IQ and achievement tests, the fact that math and reading scores are normally distributed in the population and other factors that make most testing models based on a single assessment unreliable for identifying LD (Stuebing et al. 2002).

#### Cognitive skills

According to Batsche (2006:72) another proposed marker of unexpected underachievement is uneven development of cognitive skills such as phonological awareness, rapid naming and working memory. To some extent, this position is based on the *Individuals with Disability Act* (IDEA) statutory definition, which associates LD with ‘a disorder of psychological processes’. A person might be identified as having LD if he or she showed low achievement and strengths in some aspects of cognitive processing.

This model was not encouraged by IDEA 2004, and no provisions have been made for it in the regulations. In fact, IDEA has never made routine assessment of cognitive processing part of the regulations because of the absence of evidence that these assessments provide unique information for identification and treatment planning, despite the routine use of these forms of testing in assessments of LD. Firstly, such assessments should not be used to identify children as having LD in the absence of evidence of low achievement. Thus, a child who has poor performance on a phonological awareness test, but average reading, is likely a false positive error (Torgesen 2002). *Underachievement* is unexpected in LD and poor test performance on a measure of cognitive or neuropsychological functioning should not be taken as evidence of LD unless it is linked to the achievement domain. It is also important to remember that poor performance on measures of academic achievement is most assuredly evidence of a cognitive deficit. There are few cognitive skills about which more is known than, for example, word recognition (Schlaggar & McCandliss 2007).

Pertaining to assessment as a means to identify learners with LD, Batsche (2006:72) suggests that the classroom teacher may screen for those students who are at risk of having oral expression and/or listening comprehension difficulties by referencing norms for oral expression and listening comprehension acquisition. A speech-language pathologist should be the one to assess and determine deficits in these two areas. This is not expertise most schools have, as such inappropriate methods are used. Inappropriate methods refer to assessment methods used to assess learners who do not experience learning difficulties. The standardised tests used by most teachers, for instance, provide the speech-language pathologist with valuable information regarding the student’s communication skills in specific areas. However, we must realise that standardised assessments may be one component of an assessment process. The use of non-standardised or informal assessments, dynamic assessment, behavioural and pragmatic observations in the ‘natural environment’ (outside of the classroom) as well as spontaneous and structured language sampling also provides important information that standardised tests by themselves may not.

According to Landsberg, Kruger and Nel (2005:46) the professional should not be engaged in the assessment of the learner but the focus should at all times be on assessment for learning. This means that it is important to break away from the performance-oriented perception of assessment when dealing with a learner who is experiencing a learning difficulty of some kind. It is this researcher’s observation that without an inclusive curriculum in place, teachers in the country find themselves using assessment methods that do not take into account the needs of learners with special needs in their classrooms.

### Teacher training barrier to effective teaching and learning

According to Bagree and Lewis (2013:2) teachers are often simply not trained or supported to teach children with LD, which makes these children among the most marginalised in terms of educational opportunity and attainment. National standards for teacher training can vary considerably between countries and are often inadequate. Teacher training for regular teachers also rarely prepares teachers for working in diverse classrooms and in particular does not equip them with the confidence, knowledge and skills to effectively support learners with disabilities. This is a key reason why so many children with disabilities remain out of school or excluded from the learning process within school. Bagree and Lewis (2013:4) further argue that if we are to reignite progress towards quality basic education (early childhood, primary and lower secondary schooling) for all, then regular teachers need to be prepared to meet the learning and participation needs of children with disabilities. To do this they need to be given appropriate initial training, ongoing training and professional development, and ongoing access to adequate high quality support and advice from specialist personnel.

A study by Mahlo (2011:161) reiterates that most classroom teachers indicate that they need intensive training in inclusive education so that they are able to support learners with special educational needs (SENs) in their classes. The classroom teachers were frustrated by situations that they were unable to handle, such as abuse children had experienced. Research further reveals that teachers who have not undertaken training regarding the inclusion of students with disabilities and special learning needs may exhibit negative attitudes toward such inclusion (Van Reusen & Barker 2001), whilst increased training was associated with more positive attitudes toward the inclusion of students with disabilities (Powers 2002). Training in the field of special needs education appears to enhance understanding and improve attitudes regarding inclusion (Kuester 2000). Introductory courses offered through teacher preparation programmes may sometimes be inadequate in preparing the general educator for successful inclusion (Beattie, Anderson & Antonak 1997).

Sometimes educators, often through inadequate training, use teaching styles that may not meet the needs of some of the learners. An educator may teach at a pace that only accommodates learners who learn very quickly. Alternatively, the pace and style of teaching may limit the initiative and involvement of learners with high levels of ability. What is taught or the subjects that learners are able to choose may limit the learner’s knowledge base or fail to develop the intellectual and emotional capacities of the learner. Such barriers arise when sufficient attention is not given to balancing skills that prepare learners for work (vocational skills) and skills that prepare the learner for coping with life (life skills) (Department of Education 1998:7). Some learners are excluded from certain aspects of the curriculum as a result of ignorance or prejudice. For example, learners with physical disabilities are often prevented from playing sports or are not given the opportunity to do so. Similarly, male and female learners are encouraged or pressurised to take certain subjects at school or at tertiary level according to their gender because those subjects will equip them for jobs that are stereotypically undertaken by men or women (Grossman 2004:209).

### Unqualified and underqualified teachers

According to Savolainen (2009:16) teachers play an essential role in quality education and thus the quality of an education system cannot exceed the quality of its teachers. Studies show that teachers become more willing participants in inclusion when they view themselves as competent and prepared to teach students with disabilities. Hull (2005) reiterates that training needs to continue to provide assistance with differentiated instruction and with modifying and adapting curricula to meet various students’ needs. The development of educators, service providers and other human resources is often fragmented and unsustainable. The absence of ongoing in-service training of educators, in particular, often leads to insecurity, uncertainty, low self-esteem and lack of innovative practices in the classroom (Department of Education 1998:11). This may result in resistance and harmful attitudes towards those learners who experience learning breakdown or towards particular enabling mechanisms.

Teachers and researchers often express concerns about training when discussing the abilities of teachers to cater for the diverse needs in inclusive classrooms. Loreman and Harvey (2005) argue that inclusion failed because in part, teachers were unable to meet the demands of modifying and delivering an appropriate curriculum to children with diverse educational needs because of incapacity. Barriers resulting from fear and lack of awareness may arise from the feelings of educators themselves. For example, learners with high ability are often regarded as a threat and therefore face denial of their significant abilities by unqualified and underqualified teachers.

Studies conducted post-implementation of inclusive education in Swaziland reveal that a vast majority of teachers in the kingdom’s schools are either not trained or underqualified in inclusive education; hence they feel they are inadequately prepared to teach in an inclusive classroom. According to a study by Zimba (2011:52), lack of teacher training in some inclusive schools in Swaziland has resulted in challenges in dealing with administrative requirements, as neither the administrator nor teachers were found to be competent with an inclusive curriculum. Training offered to teachers at the pioneer or pilot schools was lamented by most teachers as they felt 1 week of training was not enough to cover the vast and complex content and methods of the inclusive education field.

### Lack of support for teachers

*Support* can be defined as all activities that increase the capacity of a school to respond to diversity (Mahlo 2011:54). A supportive environment where there is collaboration among teachers, district officials, principals, parents and learner support for teachers is crucial for successful implementation of inclusive education. Support may involve a group of colleagues who are available to assist learners experiencing barriers to learning; therefore, educational support services need to be organised and the roles of all players in the implementation of inclusive education clearly defined (Calitz 2000:16).

According to Pijl and Meier (1997:9) inclusive education can only be successful if teachers elicit an attitude acceptable to all learners and when they have sufficient support and resources to teach all learners. Teachers in the kingdom of Swaziland are to a large extent lacking this support as the Ministry of Education and Training has only recently established structures for teacher support. For instance, a bachelor’s degree in inclusive education was introduced at the Southern Africa Nazarene University in the year 2012 as well as inclusive education courses in the other teacher training colleges. Whilst this is a positive step towards capacity building, a large number of teachers who are already in the field still feel they lack the skill and the tools to teach learners with diverse needs because most of them never received training in inclusive education, whilst capacity-building workshops have not been able to reach a majority of teachers in the field.

According to Fakudze (2012:74) lack of support for teachers is characterised by lack of state funding for inclusive education programmes and provision of in-service training for teachers that can empower them and so lead to a change in their attitudes towards inclusive education. Fakudze (2012) further argues that teachers upgrade themselves at their own expense on a part-time basis. Moreover, government does not reward teachers’ achievements through properly remunerating them after obtaining appropriate qualifications. In addition, the Ministry of Education and Training has failed to provide schools with specialists in areas such as braille, hearing specialists and learning difficulty specialists to mainstream inclusive schools. In a study conducted by Mahlo (2011:176) in Gauteng Province in South Africa, interviews revealed that the school-based support teams (SBST) lacked the knowledge and skills to assist learners and teachers and yet empowering the SBSTs could be one strategy to enhance the implementation of inclusive education.

### Inappropriate teaching and learning methods and support material

According to Le Fanu (2005), in terms of knowledge, teachers need to be aware of the different forms of diversity to be found among children. These include gender difference, linguistic, cultural and ethnic diversity, social–emotional diversity, cognitive and academic diversity and sensory and physical diversity. Many of these diversities are interconnected and also embedded in various contexts. For instance, it is not possible to understand the problems faced by girls in schools without considering the impact of some traditional beliefs on the ways they are regarded and treated. As Webster (2004:1) indicates, schools in Papua New Guinea can perpetuate and exacerbate repressive attitudes but schools can also be a ‘ladder of opportunity’ for girls as well as boys.

The impact of inappropriate teaching and learning methods can be demonstrated in a study conducted in a primary school in Botswana. During class observations, Mukhopadhyay, Molosiwa and Moswela (2013:5) observed that teachers were using the teacher-centred method, which did not cater for individual differences. Their lesson notes were scanty without clear evidence on how they would meet the learning needs of learners with SENs. None of these teachers employed instructional accommodation during teaching and learning. Another interesting finding was that some of the teachers preferred to use Setswana when interacting with learners with SENs during the lesson. Post-observation interviews revealed that teachers felt that learners with SENs did not comprehend well when instructed in English. The data suggested that the teachers were operating within the deficit model, which views student with disabilities as ‘incapable of learning’.

Mukhopadhyay et al. (2013:6) also observed that at a school with a long history of practising inclusive education, regular teachers collaborated well with special educators. Their teaching approaches were ideal because they employed instructional adaptations and strategies such as (1) large fonts for learners with visual impairments and (2) peer-tutoring to meet the learning needs of individuals with visual impairments. The culture of teaching at this school also emphasised team-teaching, instanced by the presence of regular and special educators who shared teaching responsibilities. The juxtaposition of these scenarios highlights the effectiveness of appropriate teaching methods against inappropriate ones.

In a study by Najjingo (2009:45) key respondents and teachers agreed that the lack of instructional materials affects the access to all-inclusive education, where learners are supported by parents 100%. This phenomenon is directly related to poor macro policy on these materials and the high costs on the open market. The critical lack of instructional materials means that though inclusive education is in place, when children with SENs lack learning aids and support appliances, their mobility is reduced and they feel inferior to their ‘normal’ peers (Najjingo 2009). They have to continuously play catch up. As a result, their pace in learning becomes slow because they are not able to hear, see or express themselves properly or because they write more slowly than other children, and learning at unfriendly facilities results in many of them failing to pass exams. It is evident in this literature that use of inappropriate teaching and learning methods and support material negatively impacts the process of implementing inclusive education.

## Research methods

The approach selected for this study was the qualitative research approach to get more information on the barriers teachers face in the implementation of inclusive education in high schools in Swaziland. The selection of this approach was based on the nature of the research problem and the characteristics of qualitative research that are applicable to this research. A case study was selected as a design for this study. According to McMillan and Schumacher (2006:398), a case study design data analysis focuses on one phenomenon that the researcher selects to understand in depth, regardless of the number of sites or participants. Data was obtained through semi-structured research interviews and documents analysis, processed and analysed through data coding, unitising and categorising, wherein the themes that emerged became the findings of the study. This research study being qualitative and a case study in nature employed social or realist constructivist methods. Social constructionism may be defined as a perspective that believes that a great deal of human life exists as it does because of social and interpersonal influences (Owen 1995:1). According to Fetterman (1998:207) knowledge is socially constructed as the researcher and the researched (teachers) interact in a natural setting. Hence, in order to investigate the research problem, teachers were interviewed and interacted with in their respective schools.

### Population and sampling

*Population* refers to a group of elements or cases, whether individuals, objects or events, that conform to specific criteria and to which we intend to generalise the results of the research (McMillan & Schumacher 2010:129). The target population of this study comprised high school teachers in the Gege branch of schools. There is a total number of three high schools in the branch. The Gege branch was selected because it is, like the rest of the country, one of the branches earmarked to mainstream inclusive education. Sampling was performed on the population of teachers from two schools selected as the case study of the research.

According to Trochim (2006), sampling is the process of selecting units from a population of interest so that by studying the same we may fairly generalise our results back to the population from which they were chosen. A researcher may use various forms of sampling techniques such as random, probability, proportional, systematic, cluster, convenience and purposive sampling, among others (Creswell 2005:204).

Purposive sampling was used in this study. This method of sampling involves the deliberate selection of a small number of information-rich cases from a larger population for in-depth study (McMillan & Schumacher 2010:399). McMillan and Schumacher (2006:401) argue that purposive sampling is used to increase the utility of information obtained from a small sample, which is the case with this research study. From a population of 60 teachers, 14 were selected: seven participants at School A and seven at School B. The teachers interviewed comprised teachers who were new in the field (with less than 1 year’s teaching experience) as well as those who had been teaching a minimum of at least 5 years. The selection of novice teachers alongside experienced individuals was purposely done because they represent different eras in teacher training. Recent graduates were selected to represent teachers who were likely to have received inclusive education training at college or university. This is in light of the fact that a 3-year Inclusive Education degree was introduced at Southern Africa Nazarene University 3 years ago (year) while more experienced teachers represented teachers who were likely to have never received pre-service inclusive education training but ought to have gotten in-service training.

### Instruments and procedures

Data used in this study was collected at the schools’ premises. Letters describing the study were sent to the teachers, who gave their written consent to the researcher. Semi-structured interviews were initiated by the interviewer, with a view to gathering certain information from the person interviewed. These were conducted face to face with individual teachers. Face to face interviews enabled the researcher to gather information about the situation regarding barriers in the implementation of inclusive education at the Gege branch of high schools. Approximately 30-minute long interviews were conducted after working hours and during teachers’ free teaching periods until all the respondents selected were interviewed. Interviews were voluntary, and respondents who participated gave the researcher their signed consent forms. The researcher used digital recording devices to record the interviews and then interviews were transcribed and coded by two independent researchers.

Interview questions were asked based on a literature review previously conducted to ascertain what other scholars have found in the same topic and the gaps thereof. The main research question the study sought to address was: What are the barriers to the implementation of inclusive education in high schools in Gege branch, Swaziland? The literature reviewed to inform the research questions included the following subtopics, inappropriate teaching and learning methods and support material, lack of support for teachers, unqualified and underqualified teachers, teacher training barriers to effective teaching and learning, inappropriate assessment procedures and curriculum delivery barriers to learning.

Semi-structured interviews and questionnaires (formatted on a five-point Likert scale) were used for data collection. Through the use of the semi-structured form of interviewing, the researchers were able to look at the way the responses were given, the tone used, facial expression, hesitation and gestures. To establish reliability, the instruments were pilot-tested with eight (four male and four female) teachers in a mainstream inclusive school in Nhlangano area. To ensure instrument reliability, the researchers used Cronbach’s alpha coefficients. The reliability of the instrument was obtained at a Cronbach’s alpha coefficient of 0.85. This instrument was deemed reliable because the acceptable Cronbach’s alpha coefficient reliability is 0.70 and above (De Vos et al. 2005). Importantly, findings from the pilot indicated that the items on the questionnaire instrument were clearly worded as there were few clarity-seeking questions and the instruments were seen as giving a satisfactory validity.

The policy documents reviewed were the Swaziland Education Sector Policy, 2011, and the teachers’ qualification registration form. The documents reviewed for this study gave information that complemented information gathered through interviews. For instance, teachers’ qualification registration forms provided evidence regarding how many of the interviewed participants had been trained and had not been trained in inclusive education.

### Data analysis

The main question was interrogated through interviews with the participants. Responses to the questions asked regarding barriers in the implementation of inclusive education were tabulated in Table 3. The responses as given by the participants culminated in the formation of three categories, which ultimately resulted in the themes or findings of the study, which are presented in Table 3. A teacher qualification register was analysed in the participating schools to investigate the level of teacher training and its impact on the implementation of inclusive education. Tables 1 and 2 are teacher profile tables describing teachers’ work experience and age.

**TABLE 1:** Profile of teachers.

Teacher	Qualification	Teaching experience	Age
A	Secondary Teachers’ Diploma	6	32
B	BA Humanities Postgraduate Certificate of Education	10	36
C	Secondary Teachers’ Diploma	15	40
D	Secondary Teachers’ Diploma	5	25
E	BA Humanities Postgraduate Certificate of Education	20	54
F	BA Humanities Postgraduate Certificate of Education	12	42
G	BA Humanities Postgraduate Certificate of Education	12	40
H	Secondary Teachers’ Diploma	10	31
I	Secondary Teachers’ Diploma BEd Inclusive Education	6	27
J	BA Humanities Postgraduate Certificate of Education	4	24
K	BA Humanities Postgraduate Certificate of Education	11	35
L	BA Humanities and PGCE	14	46
M	Secondary Teachers’ Diploma	8	37
N	BA Humanities and PGCE	17	48

BA, bachelor of arts; BEd, bachelor of education; PGCE, postgraduate certificate in education.

**TABLE 2:** Summary of teacher profiles.

Age, years (*n*)	Teaching experience, years (*n*)	Qualification (*n*)
20–29 (3)	1–9 (3)	BEd Inclusive Education (1) Secondary Teachers’ Diploma (2) BA Humanities + postgraduate certificate (1)
30–39 (5)	6–11 (5)	Secondary teacher’s diploma (3) BA Humanities + postgraduate certificate (2)
40–60 (6)	15–20 (6)	Secondary teacher’s diploma (1) BA Humanities + postgraduate certificate (3) BA Humanities and PGCE (2)

BA, bachelor of arts; BEd, bachelor of education; PGCE, postgraduate certificate in education.

### Ethical considerations

The researcher was fully aware of the ethical and legal obligations he had to the study and the participants as well. The obligations included full disclosure of the study to participants, voluntary participation of respondents, informed consent and avoiding exposing participants to risks. Researchers also have an obligation to protect the privacy of participants, hence the need to pay attention to practices such as anonymity, confidentiality and appropriate storage of data (McMillan & Schumacher 2010:121).

The authors adhered to research ethics and the University of South Africa provided clearance. All stakeholders including participants consented by signing consent forms.

## Results of the research

Participants were male (10) and female teachers (4), all qualified high school teachers, employed by the Ministry of Education and Training on a permanent basis in their schools. Some teachers were diploma holders whilst others were degree holders, and their teaching experience varied from 4 to 20 years. Participants have been represented by letters A to N under the teacher section of Table 1. The names of the teachers are known but letters have been used to protect their identities and ensure confidentiality.

The individual teacher profiles were grouped to show age, experience and qualifications. The aim was to summarily give the number of teachers with relevant qualifications in inclusive education. The summary of teacher profiles is presented in Table 2.

All interviews with participants were conducted by the researcher during times that were suitable and comfortable to the participant teachers. Other participants were interviewed on weekends and after school hours when they had free time to answer the research interview questions.

### Data coding

According to Lee (2007:3), for the first step of the data analysis, the researcher has to read and reread, writing down the emerging categories in a form of a paraphrase, phrase, heading or label that describes what the respondents are trying to say and what the researcher thinks of as important. This process is called ‘data coding’ (Mertler 2006:3). Coding requires the researcher to reduce the volume of information collected in order to identify and organise the data into important patterns and/or themes; hence the coding of the collected data became the first step to data analysis in this study.

To reduce the impact of researcher bias, two independent researchers were engaged in the process of data coding and the themes that emerged from both were taken as the findings of the study. Below are the findings presented in tubular format as coded by both independent researchers. The themes are further deliberated on in the discussion section of this research study. Table 3 presents the responses to the question asked regarding barriers in the implementation of inclusive education that participants identified as realities in their respective schools.

**TABLE 3:** Units, categories and themes that emerged during data analysis.

Units	Categories	Themes
Barriers to the implementation of inclusive education		
Big numbers of learners in classrooms. Overcrowded classes. Teacher cannot cater for the needs of all learners.	Teachers have the problem of high numbers of learners.	Non-inclusive curriculum, high numbers of learners, lack of resources and teachers’ lack of competency are barriers to the implementation of inclusive education.
Completion of syllabus is slowed down by workload. Curriculum is not inclusive. Lack of resources and equipment for the disabled.	Lack of resources and non-inclusive curriculum.	
Infrastructure not catering for the disabled. Teachers lack competence to deal with learners experiencing challenges. Teachers are not adequately trained in dealing with learning challenges. Teachers’ inability to identify learners with learning challenges. Teachers have negative attitude towards teaching learners with disabilities and learning challenges.	Teachers do not have competence in dealing with learners experiencing learning challenges.	
Training in inclusive education and identifying learners with learning challenges		
Not trained at all. Have not received training. Not trained at college. Not at all. Not trained in my teaching career.	Teachers were not trained in inclusive education.	Teacher training in inclusive education is inadequate, and training in identifying learners with challenges is inadequate, not properly structured.
Just introduced to inclusive education. Just taught about the meaning of inclusive education at college.	Training received by teachers was not intensive.	
Touched on it in my psychology studies. I am now studying inclusive education at university. I was given an overview of inclusive education in my guidance and counselling studies. Taught about dealing with learners with vision problems. Taught to be observant of strange signs in children’s behaviour. Not trained, just learnt informally. Not sufficiently trained. Just trained in psychology class. Trained to observe learner behaviour. Identify them without training.	Content learned for identification of learners with challenges is shallow.	

### Coded data: Researcher 1 analysis

#### Summary of themes that emerged from the study

Table 3 presents the findings of this study that highlight major factors that were found to be barriers to the implementation of inclusive education in schools by teachers. These include the following.

**Non-inclusive curriculum:** The curriculum is considered to be non-inclusive if it does not take into consideration activities teachers and learners must to do because of learning challenges and barriers that may be present in inclusive classrooms. This was found to be true of the curriculum of mainstream schools in Swaziland and this fact has been further elaborated on in the ‘Discussion of findings’ section.

**Big numbers of learners:** The policy of the Ministry of Education and Training in Swaziland is that one teacher should teach 40 learners per classroom. Teachers complain that whilst this is practical for so-called normal school learners, it is much more challenging for the inclusive classroom where a teacher has to spend more time on one learner and draw up Individualised Education Programmes for pupils with learning challenges.

**Lack of resources:** The units in Table 3 highlight that teachers asserted there was a lack of resources and equipment (assistive devices) for disabled learners in the schools where they worked. There was also a lack of teachers and yet in inclusive schools they are an essential resource.

**Teachers’ lack of competence as barriers to the implementation of inclusive education:** The teachers’ responses in Table 3 further divulge that teacher training in inclusive education and training in identifying learners with challenges was inadequate among the teachers and not properly structured. One document reviewed was a teacher profile document that contained teacher information and their qualifications from both participating schools. This document showed that in schools with 61 staff members there was one teacher holding a certificate or any qualification in inclusive education, with one teacher still studying towards attaining her inclusive education bachelor’s degree.

**Lack of clear government implementation matrix policy:** The other policy document reviewed was the Swaziland Education Sector Policy of 2011. Section 6.3 of this policy calls for the mainstreaming of inclusive education. However, the section is not clear on how this shall be implemented, funded and supported. Moreover, the participating schools were found to be without their own policies adopted from this policy on how inclusive education was to be mainstreamed. The Inclusive Education: Responses, Challenges and Prospects for the Kingdom of Swaziland Report notes that in some quarters inclusive education is still narrowly defined and associated with disability only and there is inadequate monitoring and evaluation of inclusive education at national level. This finding by the report is consistent with responses from some teachers who admitted to having a negative attitude towards learners with disabilities and those having learning difficulties. Whilst monitoring at national level is overseen by one inspector in the Ministry of Education and Training, the country remains without a regional inspectorate for inclusive education.

### Coded data: Researcher 2 analysis

The second researcher transcribed the audio interviews with participants of the study and the findings that emerged were listed and titled ‘Themes’.

#### Themes: Barriers to the implementation of inclusive education

- High numbers of learners.
- Curriculum is not inclusive.
- Identification of learners with learning challenges.
- Teachers lack competence to support learners with challenges.
- Teachers not adequately trained in dealing with learners with learning challenges.
- School buildings lack assistive equipment.
- Non-inclusive curriculum.
- Inadequate attention to learners with challenges because of other teaching activities.

## Discussion of findings

The data gathered during this research study was coded by two independent researchers and the primary researcher analysed both sets of themes. The researcher observed that, to a large extent, the themes, which are the findings of the study, are largely similar, although they were coded separately. The participants’ responses to questions aimed to help the researcher ascertain what the barriers are to the implementation of inclusive education in schools in Gege branch, Swaziland. The themes presented are further discussed in detail in the following.

### Non-inclusive curriculum, big numbers of learners, lack of resources and teachers’ lack of competence are barriers to the implementation of inclusive education

A respondent had this to say:

‘Teaching in a class with big numbers and having learners who experience learning challenges does slow down the teaching and learning process. At the end of the day syllabuss may not be completed at schools.’ (Respondent A, female, 32 years old)

Teachers viewed inclusive education efforts as being stifled by the large number of learners they were currently teaching in their classrooms. Teachers’ argument in this regard was that it becomes very difficult to cater to every child’s individual needs in a big classroom setup and still be able to meet other demands such as test schedules, piles of marking and constant evaluation. It was also argued by teachers that considering extracurricular activities in schools, inadequate attention is offered to learners with challenges.

Most teachers complained that a majority of their classes had more than 40 students per classroom, which is more than the official student–teacher ratio stipulated by the Ministry of Education and Training. To this end it can be argued that indeed classrooms were overcrowded as some teachers asserted.

Other teachers observed that the large numbers of students per class can perhaps be attributed to inflexibility of the curriculum. A respondent reiterated that:

‘The curriculum used itself, is not inclusive as it doesn’t take into consideration activities teachers and learners ought to do due to learning challenges and barriers that may be present in inclusive classrooms.’ (Respondent C, male, 25 years old)

It was felt not appropriate that the Ministry of Education and Training was mainstreaming inclusive education but still using a curriculum that was not designed to be inclusive. This argument by teachers seems valid because a curriculum needs to take into consideration teaching methods, lesson planning and lesson structures, as well as assessment methods. Because the current curriculum does not recognise the diversity of learners and their needs in the classroom, then indeed it does hinder efforts towards inclusion.

Another respondent noted the lack of facilities in the school as a challenge in implementing inclusive education in his school. A respondent observed:

‘[*T*]he school lacks facilities to cater for learners with disabilities whilst changes in school buildings have not been done to accommodate learners with physical disabilities such as learners in wheelchairs.’ (Respondent B, male, 36 years old)

However, Respondent H (female, 31 years old) submitted that lack of training in inclusive education makes it hard for a teacher to be in a position to support a learner with learning challenges even if the teacher can identify a learner’s problem area. She further noted that a learner can be identified to be having a learning challenge but if parents do not have the resources it becomes difficult to effectively help a child. For instance if a child has an eye problem requiring reading glasses and the parents cannot afford them, it becomes problematic. The child does not learn for long periods and thus misses out on a lot of subject matter. Other teachers felt that parents did not support them enough as well, because sometimes a teacher may be able to identify a learner to be having eyesight problems, for instance, which can be addressed by taking a child to an eye specialist who will recommend appropriate intervention. In such cases teachers complained that parents were usually unable to pay for the child’s medical bills, which may mean the child would not be helped for a prolonged period, thus resulting in the child’s eye problems becoming severe; hence the same effect would occur on his or her learning.

Poverty-stricken communities are also poorly resourced communities, which are frequently characterised by limited educational facilities, large classes with high pupil–teacher ratios, inadequately trained staff and inadequate teaching and learning materials. Such factors raise the likelihood of learning breakdown and the inability of the system to sustain effective teaching and learning. Learners from families where one or more of the breadwinners are unemployed or poorly paid are also more likely to leave school as soon as possible to go out to work to supplement the family income. This perpetuates the cycle of limited skills with fewer work opportunities, increased likelihood of unemployment or poorly paid work and, thus, ongoing poverty and exclusion (Department of Education 2008).

Respondent D (male, 25 years old) commented that he was unable to identify learners facing learning challenges in his classroom. According to Gwala (2006:3), often teachers who are unable to identify learning difficulties in learners have the tendency to believe that learners are being difficult or deliberately disturbing learning; hence in some cases teachers ended up punishing learners who in fact needed help. According to Fakudze (2012:71), in such cases learners end up dropping out of school because of punishment and lack of support from their teachers.

One of the respondents commented that in the schools there was a problem of resources. She said:

‘For instance, there is no way we can teach learners with sight problems because we do not have braille machines so such teaching materials or resources we still do not have. Secondly, the school buildings are still not adjusted to accommodate learners with disabilities who may require assistive equipment and specially designed buildings to help them move around the school.’ (Respondent I, male, 27 years old)

### Lack of support for learners with challenges

South Africa, in an effort to support the implementation of inclusive education at foundation phase, introduced specialist teachers called learning support teachers (LSTs). Many teachers in that country did not have the benefit of being trained to teach learners who experience barriers to learning; hence most find it difficult. According to Mahlo (2011:5) these expectations have been employed in the foundation phase to fill that gap and assist classroom teachers. The teachers in our study argued that whilst Swaziland does not have such a crucial structure as the LSTs, the in-service training department in the Ministry of Education and Training is believed to be the equivalent. The in-service department is known for its role in training teachers who are already in the field, particularly to boost their capacity when new programmes are introduced. In view of this, teachers felt that the in-service department’s inactivity where inclusive education was concerned was the reason for the lack of support for learners with learning challenges.

### Lack of resources and appropriate infrastructure for disabled learners

Most schools in Swaziland are not accessible to disabled learners such as the blind and deaf. Observation by teachers interviewed for this study was that the infrastructure of schools in the Gege branch is not accessible for learners with disabilities, especially those in wheelchairs. Against this background, the government of Swaziland has a project to modify the infrastructure of schools to increase physical access for children with special needs. However, teachers in the Gege high schools indicated that infrastructural development remains a concern. Some schools were constructed on sloping ground. This is a huge challenge for blind pupils and pupils in wheelchairs. Teachers also noted that the government only delivered mobile classes in primary schools and these classes have no provisions for blind pupils or pupils in wheelchairs. Teachers felt that the school infrastructure must be upgraded to accommodate pupils who are blind and are using wheelchairs.

It was also observed by teachers that whilst government policies advocate that every learner should attend schools that are in their neighbourhood, some disabled learners may not be able to access education there. It was argued that the high schools in Gege did not have resources such as braille equipment and the teachers were not competent in reading and writing braille; neither were they literate in sign language.

### Teacher training in inclusive education is inadequate and identifying learners with learning challenges is inadequate and not properly structured

Teachers were asked how they were trained in inclusive education. From their responses it was clear that not all teachers were trained at tertiary level or at in-service level. There is evidence that even those who were trained were perhaps not adequately trained. The following are some of their comments:

‘I can say I haven’t received any training in inclusive education.’ (Respondent A, female, 32 years old)

‘I wasn’t trained at college in inclusive education. What happened was that we were introduced to inclusive education, simply the definition, just what it is about. We never learnt content in details.’ (Respondent B, male, 36 years old)

‘Well, I cannot say I was trained but as part of my training at college there was a topic we did on inclusive education. It was under another subject and lasted one or two months.’ (Respondent J, male, 24 years old)

‘Not at all. As part of my training at the University of Swaziland we touched on some Millennium Development Goals, which touched on the need for inclusivity in all schools in the country, but we never touched on topics on inclusive education.’ (Respondent K, female, 35 years old)

This notion is shared by Bagree and Lewis (2013:2), who claim that teachers are often simply not trained or supported to teach children with LD, which makes these children among the most marginalised in terms of educational opportunity and attainment. This is affirmed by responses from most teachers interviewed in this study.

These responses by the teachers clearly show that teachers do not feel prepared enough to work with learners who have diverse learning needs. Hence, even those who did receive some degree of training complained that their training was not enough to prepare them to work in inclusive classrooms. For instance:

‘Barely. I only got to know about inclusive education as just an overview during one of my guidance and counselling classes at the University of Swaziland.’ (Respondent G, female, 40 years old)

This shows that there is a serious need to train teachers in the sampled schools on inclusive education methods. This is evidence that a large population of teachers in schools today have not been trained in inclusive education matters.

Teachers were further asked how they were trained to identify learners experiencing learning challenges in their classrooms. Various responses were given in response to this question but there were mixed feelings as a good number of teachers responded that they were not trained in light of them not having been trained in inclusive education at tertiary level. Other teachers alluded to the fact that identifying learners experiencing learning challenges is a skill that generally all teachers obtain during teacher training at teacher training institutions, regardless of whether the course a teacher is doing is inclusive education related or not.

‘I wasn’t trained but as a teacher you must be very observant, maintain eye contact so that you can be able to spot a learner with challenges. Being observant puts you in a position to identify and help learners experiencing learning challenges.’ (Respondent C, male, 25 years old)

‘I didn’t receive any training in inclusive education. Under educational psychology we did touch on inclusive issues but I still feel that was not enough training in the subject.’ (Respondent E, male, 54 years old)

‘Of course I was trained in identifying learners experiencing learning difficulties by observing how a learner writes, how they look at what is written on the board. Basically you have to figure it out yourself when looking at the learner’s behaviour that a learner might be experiencing challenges, then you can approach that learner and intervene in a way that addresses the challenge a learner has.’ (Respondent H, female, 31 years old)

Through responses from educators on this question, it is evident that indeed many teachers maintained that they were not trained in identifying learners with challenges. Teachers argued that they were not trained in inclusive education. Hence, they were not taught how to identify learners experiencing barriers to learning. However, closely looking at teacher’s responses, the researcher can argue that it is imperative that even those teachers who claim to have no background in inclusive education would have received training in the identification of learners experiencing learning challenges. According to Du Toit (1997), observation is the basic skill that teachers should master in order to identify learners with barriers to learning successfully. Having interacted with a lot of literature on identifying learners with learning challenges, the researcher acknowledges that indeed there are many methods, some of which are psychological and scientific, that can be used to identify learners experiencing learning problems.

### Lack of clear government implementation matrix policy

Among the documents reviewed was a teacher profile document, which contained teacher information and their qualifications. This document showed that in schools with 61 staff members there was one teacher that had a certificate or any qualification in inclusive education. One other teacher was still studying towards obtaining her bachelor’s degree in Inclusive Education. This highlighted the fact that teacher training is a very important factor towards achieving the desired competencies for the implementation of inclusive education to mainstream schools in Swaziland. The researcher also observed that negative attitudes by teachers towards teaching in inclusive classrooms are a barrier emanating from the fact that a majority of the teachers interviewed received inadequate training in inclusive education.

The other policy documents reviewed were the Swaziland Education Sector Policy of 2011 and the Inclusive Education: Responses, Challenges and Prospects for the Kingdom of Swaziland Report of 2011. The Swaziland Education Sector Policy (2011:13) states it as one of its policy goals that all attitudinal and physical barriers to inclusive education shall be removed in public, private and other schools and institutions. This research study has revealed through teachers’ responses that the barriers that the Ministry of Education and Training seeks to eliminate exist in schools today in many forms. It also transpired during the review of these documents that whilst the Ministry of Education and Training has policies in place to enforce implementation of inclusive education, schools do not have their own policies regarding the same adapted from the main government policies.

## Conclusion

The aim of this research study was to identify barriers that have affected the implementation of inclusive education in mainstream schools in Swaziland, a case study on the Gege branch of schools. The prerogative of inclusive education is to serve the needs of all learners and to ensure that they reach their optimum potential, whilst seeking to include parents and communities in this process. Achievement of this aim not only fulfils the constitutional education obligation the country has to its citizenry, but it also increases the literacy rate and much-needed skills and labour need for growth and sustainability.

While Swaziland has promulgated sound policies to allow for effective mainstreaming of inclusive education, barriers such as a non-inclusive curriculum, high numbers of learners, lack of resources and teachers’ lack of competence have emerged as challenges. To this end it is advisable for the National Curriculum Centre of Swaziland to redesign the country’s curriculum to accommodate learning needs for children with different abilities and diverse learning needs. Also, establishment of district-based support teams to liaise with schools in teacher training and inclusive education support matters in schools could help eliminate barriers facing implementation of inclusive education at Gege branch schools in Swaziland. Also, there is a need for schools to create their own implementation matrixes with regard to facilitating implementation of inclusive education policies in order to breach the disconnect that currently exists between national policy and actual practice of it.
